# Proof of concept of using a membrane-sensing peptide for sEVs affinity-based isolation

**DOI:** 10.3389/fbioe.2023.1238898

**Published:** 2023-08-11

**Authors:** Beatriz Benayas, Joaquín Morales, Alessandro Gori, Alessandro Strada, Paola Gagni, Roberto Frigerio, Carolina Egea, Pilar Armisén, Marina Cretich, María Yáñez-Mó

**Affiliations:** ^1^ Agarose Bead Technologies (ABT), Torrejon de Ardoz, Spain; ^2^ Department Biología Molecular, Universidad Autónoma de Madrid, IUBM, Centro de Biología Molecular Severo Ochoa, IIS-IP, Madrid, Spain; ^3^ Consiglio Nazionale delle Ricerche, Istituto di Scienze e Tecnologie Chimiche “Giulio Natta” (SCITEC), Milan, Italy

**Keywords:** extracellular vesicles, peptide, isolation, affinity, agarose beads

## Abstract

**Introduction:** One main limitation in biomarker studies using EVs is the lack of a suitable isolation method rendering high yield and purity samples in a quick and easily standardized procedure. Here we report an affinity isolation method with a membrane-sensing peptide (MSP) derived from bradykinin.

**Methods:** We designed a protocol based on agarose beads carrying cation chelates to specifically bind to the 6His-tagged membrane-sensing peptide. This approach presents several advantages: 1) cation-carrying agaroses are widely used and standardized for His-tagged protein isolation, 2) the affinity protocol can be performed in small volumes, feasible and manageable for clinical routine and 3) elution with imidazole or EDTA allows a gentle and easy recovery without EV damage, facilitating subsequent characterization and functional analyses.

**Results:** The optimized final procedure incubates 0.5 mg of peptide for 10 min with 10 µL of Long-arm Cobalt agarose before an overnight incubation with concentrated cell conditioned medium. EV downstream analyses can be directly performed on the agarose beads adding lysis or nucleic-acid extraction buffers, or gently eluted with imidazole or EDTA, rendering a fully competent EV preparation.

**Discussion:** This new isolation methodology is based on the recognition of general membrane characteristics independent of surface markers. It is thus unbiased and can be used in any species EV sample, even in samples from animal or plant species against which no suitable antibodies exist. Being an affinity method, the sample handling protocol is very simple, less time-consuming, does not require specialized equipment and can be easily introduced in a clinical automated routine. We demonstrated the high purity and yield of the method in comparison with other commercially available kits. This method can also be scale up or down, with the possibility of analyzing very low amounts of sample, and it is compatible with any downstream analyses thanks to the gentle elution procedure.

## Graphical Abstract

**Figure:**
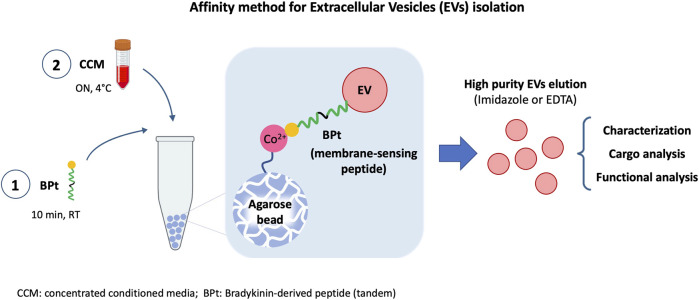

## Introduction

The interest in extracellular vesicles (EVs) has steadily raised in the last years due to their relevance in physiological and pathological conditions as mediators of intercellular communication. They are secreted by eventually every type of cell and can transfer bioactive molecules such as proteins, lipids and nucleic acids (Yáñez-Mó et al., 2015). Their isolation from biological fluids points them as really useful for new biomarker discovery and other clinical approaches (Fais et al., 2016; Gutiérrez-Fernández et al., 2021).

The most usually employed techniques for EVs isolation are differential ultracentrifugation (dUC), flotation in a density gradient (DG-UC), size exclusion chromatography (SEC) or precipitation based-methods and affinity methods. None of them is an ideal method, with advantages and disadvantages that have been previously reviewed (Monguió-Tortajada et al., 2019; Clos-Sansalvador et al., 2022). The selection of a given isolation method is determined by several factors as sample origin and amount, costs, equipment required and downstream analyses.

The classical and still the most employed method for EVs isolation is dUC (Théry et al., 2006; Gardiner et al., 2016). It provides high EVs yields, but with low purity, as protein aggregates often coprecipitate with EVs. It is time-consuming and depends on ultracentrifuge availability which hampers its use in many clinical settings.

DG-UC separates particles depending on their density. It provides EVs samples with higher purity, being able of eliminate protein aggregates and the majority of lipoproteins, with the exception of some subpopulations that present similar density to EVs (Karimi et al., 2018). However, it renders low EV yields and, as happened with dUC, it is highly time-consuming and requires specific equipment.

SEC allows the separation on particles depending on their size. It provides intermediate EVs yields with higher purity, as it efficiently eliminates soluble protein in samples. As DG-UC, SEC may allow the elimination of the majority of lipoproteins, except subpopulations like chylomicrons, whose sizes overlaps with EVs (Karimi et al., 2018), and in some configurations very abundant LDL lipoproteins in blood-derived samples (Benayas et al., 2023). This technique is easy, cheap and does not require special equipment. It also allows the functional interrogation of separate components in the sample (EVs, lipoproteins, soluble factors). However, translation to clinic could be still difficult when processing several samples and has yet some room for improvement.

Precipitation based-methods are easier, cheaper and quicker than all the previously mentioned techniques (Ludwig et al., 2018; Konoshenko et al., 2021). However, EVs samples obtained by this method present the highest levels of non-EV components contamination.

Finally, affinity methods allow for a higher specificity in EVs isolation, as they are based in the recognition or binding to molecular components present on EV surface, decreasing the level of contaminants in the isolated EVs. Moreover, these methods are usually relatively rapid, easy and feasible for clinical rutinary analysis. There are different types of affinity isolation methods, whose advantages and disadvantages have been previously reviewed (Gaillard et al., 2020; Ströhle et al., 2022; Xu et al., 2022).

Among affinity methods, those using antibody recognition and capture of membrane proteins on the surface of EVs are the more common (Campos-Silva et al., 2019). They have been demonstrated to be useful for the isolation of EVs with low levels of contamination from biofluids (Tauro et al., 2012) and for the direct detection of pathological biomarkers (Campos-Silva et al., 2021). The main disadvantages of this method are that it requires very harsh methods to elute EVs from antibodies that can damage EVs and affect their downstream use; and that antibodies could also be present in the eluted sample, which would also interfere with some downstream analyses or functional studies (Tkach et al., 2018). Besides, of course, they are dependent on the availability of good antibodies against EV markers which may be challenging for some animal models, plants, etc.

Approaches like aptamer based-isolation have emerged to overcome some of these disadvantages (Wu et al., 2020). Aptamers are small oligonucleotides that also specifically recognizes membrane proteins on EVs with a gentler elution of the sample, maintaining EV function, and they are more stable and easier to prepare.

For both antibody and aptamer-based techniques, tetraspanins have been the most usual target employed for EV capture. This fact raises another drawback of these approaches, that is that they would always render a selection of EVs subpopulations depending on the surface expression of that marker on EVs (Kowal et al., 2016). To date, no universal and homogeneously distributed protein EV marker has been reported that would allow the isolation by protein-targeted affinity methods of all EVs particles.

Methods based on membrane characteristics of EVs are a good option to solve this problem. Some studies have described that EVs present phosphatidylserine (Llorente et al., 2013) in their outer membrane (Hugel et al., 2005) and this property can be used for EVs isolation using TIM4 protein for its recognition (Nakai et al., 2016; Yoshida and Hanayama, 2022). However, some studies have demonstrated that PS-negative EVs are more abundant in biofluid samples than positive ones (Matsumoto et al., 2021).

Another good option is the use of membrane-curvature sensing peptides. Peptides that can specifically bind to highly curved membranes have been described (Saludes et al., 2013) and efficiently used for the analysis of small EVs (sEVs) (Gori et al., 2020) and the isolation of lipid nanovesicles (Salerno et al., 2022). The amphipathic characteristics of these peptides allow the recognition of negative charges on sEVs membrane by electrostatic interactions, and then lately specifically recognize defects (gaps) induced by the high curvature of sEVs membranes. The use of this kind of peptides would maybe in part solve the problem of the absence of a universal EV marker, while it would have all the advantages of affinity methods for a feasible isolation method in a clinical routine. Moreover, peptides in comparison to proteins are more stable and easier to produce, which also highlight their promising future use for EVs isolation. Therefore, in this work we have tested and demonstrated the isolating capacity for EVs of this peptide optimizing the conditions for a simple affinity method.

## Materials and methods

### Cell culture and EVs production

Human melanoma cell line SK-MEL-147 was cultured in DMEM supplemented with 10% heat-inactivated FBS, penicillin (100 U/mL) and streptomycin (100 μg/mL), at 37°C in a 5% CO_2_ atmosphere. For EV production, 1.6 × 10^6^ cells were grown in p150 plates for 6 days in DMEM supplemented with 5% EV-depleted FBS until they reached confluence. EV depletion from FBS was performed by ultracentrifugation at 120,000 g for 16 h.

Conditioned media was centrifuged for 5 min at 500 g and 30 min at 3,200 g to eliminate cells, cellular debris and apoptotic bodies. CM from 1 p150 plate (20 mL) was then concentrated (concentrated conditioned media: CCM) using Amicon Ultra-15 filters (100K, Millipore, Billerica MA) to a final volume of 400 μL, which was subsequently used for EV isolation.

### Membrane sensing-peptide and EV isolation by affinity chromatography

Bradykinin-derived peptide (BP) previously employed in (Gori et al., 2020) was modified adding a 6 His-tag (6xHis-RPPGFSPFR) to allow divalent cation binding, enabling stable anchoring to resin supports. Tandem (BPt) or branched (BPb) molecular constructs were used as indicated, which are composed of 2 or 4 BP repetitions, respectively (see section below and Figure 4A). Agarose resins employed were customized by Agarose Bead Technologies (ABT) to improve the extern interaction of cations in the surface of the bead. The information of the different agarose resins employed are included in Table 1.

**TABLE 1:** Resins information.

Resin name	Density of chelate groups	Length of arm between agarose and chelate
High density nickel QTS1	43 μmols Ni^2+^/ml	4 atoms
High density cobalt QTS2	42 μmols Co^2+^/ml	4 atoms
Low density copper QTS3	16 μmols Cu^2+^/ml	4 atoms
High density cobalt QTS2BL	20 μmols Co^2+^/ml	12 atoms

For each experiment, volumes, concentrations and incubation times are indicated in results and figure captions. In brief, for most experiments 0.5 mg of BP were coupled to 10 µL of agarose by incubation at RT in 0.2 mL tubes. After peptide binding, agaroses were washed twice with 100 µL of PBS to eliminate unbounded peptide and incubated with 100 µL of concentrated condition media for EVs binding over night at 4°C, under rotation. Unbound material was discarded, and resins were washed 5 times with 100 µL of PBS. All washes were performed pelleting the agarose at 1,000 rpm for 30 s.

For downstream dot blot analysis, resins were lysed with Laemmli buffer in a total volume of 30 µL (including resin volume). For EVs characterization by NTA, MET, western blot and for SiMoA assay EVs were eluted with 100 µL of filtered PBS containing 0.5 M imidazole (Sigma) (EVs characterization by NTA, MET, western blot). For uptake analysis by flow cytometry, EVs were eluted with 100 µL of filtered PBS containing 0.5 M imidazole or 100 mM EDTA, as indicated.

### EV isolation by Size Exclusion Chromatography (SEC)

Empty columns with both upper and under cellulose frits were packed with 10 mL of 2% BCL agarose (Agarose Bead Technologies). Columns were washed with 2 volumes of filtered PBS before SEC. Concentrated condition media from 1 p150 (400 µL) was loaded and 25 fractions of 500 µL were collected by gravity elution using PBS as elution buffer.

### Dot blot and western blot analysis

For dot blot analysis, agarose-peptide-EVs resins were lysed with Laemmli buffer in a total volume of 30 µL (including resin volume) and heated for 10 min at 65°C. 1 μL from the different samples were directly spotted on a nitrocellulose membrane (GE Healthcare) and let to dry.

For SK-MEL-147 whole cell lysates, confluent cells were washed with PBS and lysed in TBS/1% Triton supplemented with protease inhibitors (Roche). Protein concentration was measured with Pierce™ BCA protein assay kit (Thermo Scientific, 23225).

For western blot analysis, EVs isolated by affinity chromatography were eluted from the resin with 100 µL of filtered PBS containing 0.5 M imidazole, lysed with Laemmli buffer and heated for 5 min at 96°C. 2·10^9^ isolated EVs or the indicated protein content of total lysates from SK-MEL-147 cell line were loaded in polyacrylamide SDS-PAGE gels. Electro-transference of proteins to nitrocellulose membrane (GE Healthcare) was carried out using wet electroblotting in TRIS-Glycine buffer with 20% of methanol, during 1.5 h at 300 mA. Both dot blot and western blot membranes were blocked with 5% skimmed-milk in TBS, 0.1% Tween-20 for 30 min.

Antibodies anti-CD81 (5A6; kindly provided by Dr S Levy, Stanford, USA), anti-histidine (H1029, Sigma-Aldrich, diluted 1:500) and anti-Apolipoprotein B (Calbiochen, 178467, diluted 1:500) were used for EV, peptide or LPP detection in dot blot, respectively. Other antibodies against EVs markers were also used in western blot analyses for EV detection: anti-CD9 (VJ/1.20; hybridoma supernatant, not diluted), anti-CD63 (Tea3.10; hybridoma supernatant, not diluted) (Yáñez-Mó et al., 1998), anti-TSG101 (Genetex, GTX118736, diluted 1:1,000), anti-Syntenin-1 (Synaptic systems, 133003, diluted 1:1,000), anti-ARF-6 (Sigma, A5230, diluted 1:1,000) and anti-flotillin (BD Biosciences, 610821, diluted 1:1,000). For non-EV markers detection, we employed anti-VDAC1 (Abcam, ab154856) and anti-calnexin (Enzo, ADI-SPA-865-F), both diluted 1:1,000. As secondary antibodies conjugated with HRP, we used anti-mouse (Thermo Fisher Scientific, 31430), anti-rabbit (Thermo Fisher Scientific, 31460) and anti-goat (Sigma, SAB3700316, diluted 1:10,000).

Membranes were revealed with Super Signal West Femto HRP substrate (Thermo Scientific). A 4,000-mini system (General Electrics) was used to acquired images, which were lately processed with Fiji ImageJ.

### Comparison with commercial kits

EV from conditioned media (CM) were isolated by BPt agarose beads as described above. For SEC by IZON kit, 150 µL of concentrated CM (45x) were loaded onto a SEC column (IZON qEV 70 Legacy). Fractions of 200 µL each were collected; the largest vesicles amount was found in fraction 7, according to manufacturer protocols suggestion. Polymer precipitation was performed by adding ExoQuick Reagent (ExoQuick) to 5 mL of 1x CM and incubating overnight at 4°C according to manufacturer instructions. Then precipitate vesicles were recollected by centrifugation and resuspended in fresh PBS. Bead immunocapture was performed by using commercially available streptavidin-beads (Invitrogen, Exosome-Streptavidin Isolation/Detection Reagent) functionalized with pan-tetraspanin biotinylated antibodies (CD9, Ancell, clone SN4/C3-3A2, CD63, Ancell, clone AHN16.1/46-4-5, and CD81, Ancell, clone 1.3.3.22). One test was performed using immune-capturing only, according to manufacturer suggestion (0.2 mL of 1X CM), another test was performed coupling the bead based immune-capture sample to 0.2 mL of EVs previously concentrated by polymer precipitation (ExoQuick) obtained from a starting volume of 9.5 mL of 1x CM. All incubations were performed according to manufacturer instructions. Vesicles were released from beads in 0.1 mL glycine 0.2 M, pH 2.4. For ExoSpin kit 1 mL of 1x CM sample were treated according to manufactures instructions. For MagCapture™ Exosome Isolation Kit PS (Fujifilm) 9 mL of 1x CM were incubated on beads and vesicles were released according to manufacturer instructions.

### SiMoA assay

SiMoA assay was run according to previously devised protocols (Frigerio et al., 2022). Briefly, beads were conjugated according to Quanterix Homebrew kit instructions using the recommended buffers to pan-tetraspanin antibodies (anti-CD9, Ancell, clone SN4/C3-3A2, anti-CD63 antibody, Ancell, clone AHN16.1/46-4-5 and anti-CD81 antibody Ancell, clone 1.3.3.22). Samples were analyzed with three-steps assay according to manufacturer instructions. 0.1 mL of each sample was diluted 1:4 in a diluent and placed in a well; after that a capture step with conjugated paramagnetic beads was performed. To detect captured EVs a mixture of biotinylated anti-tetraspanin antibodies was used (anti-CD9, Ancell, clone SN4/C3-3A2, anti-CD63 antibody, Ancell, clone AHN16.1/46-4-5 and anti-CD81 antibody Ancell, clone 1.3.3.22). Detection was allowed by streptavidin-β-galactosidase (SBG), which acts on the fluorogenic substrate resorufin β-D-galactopyranoside (RGP). First, a calibration curve was produced by analyzing serial dilutions of the 45x CM sample as quantified for particle concentration by NTA analysis. For the recovery yield calculation, the AEB (Average Enzyme per Bead) raw data provided by the instrument for each sample were first interpolated in the calibration curve to provide a particle concentration, which was then normalized by the volume of the starting material.

### Nanoparticle tracking analysis (NTA)

The number of particles and the size distribution of EVs after SEC or affinity isolation were analyzed with NanoSight NS300 (Malvern Instruments Ltd., Malvern, United Kingdom), equipped with a 532 nm laser. EV-enriched fractions were diluted 100 times for the analyses. Results were analyzed with NTA 3.0 software.

### Transmission electron microscopy (TEM)

EVs isolated by SEC or affinity were diluted by half in filtered PBS, adsorbed on carbon-coated nickel grids and contrasted with uranyl acetate. Sample visualization and image acquisition were performed in a transmission electron microscopy JEM1400 Flash (Jeol). The size and morphology of isolated EVs were analyzed with TEM Exosome Analyzer software (Kotrbová et al., 2019).

### Analysis of EV uptake

EVs obtained by BPt affinity method (and SEC) were labelled with Alexa 633 C5 Maleimide (Invitrogen) at 4°C ON. Maleimide excess was removed by SEC in columns of 2 mL using 2BCL agarose. Positive EVs fractions were pooled, and the number of particles was determined by NTA. 25,000 EVs per cell were incubated with 100000 SKMEL-147 cells for 30 min, 1 h or 2 h (as indicated) in regular culture conditions to allow EV uptake. Cells were washed with PBS, detached with trypsin and washed again with PBS. EV uptake was measured with a FACSCantoII flow cytometer and results analyzed with FlowJo software.

## Results

### Optimization of the coupling of membrane-sensing peptide to agarose resins

**FIGURE 1:**
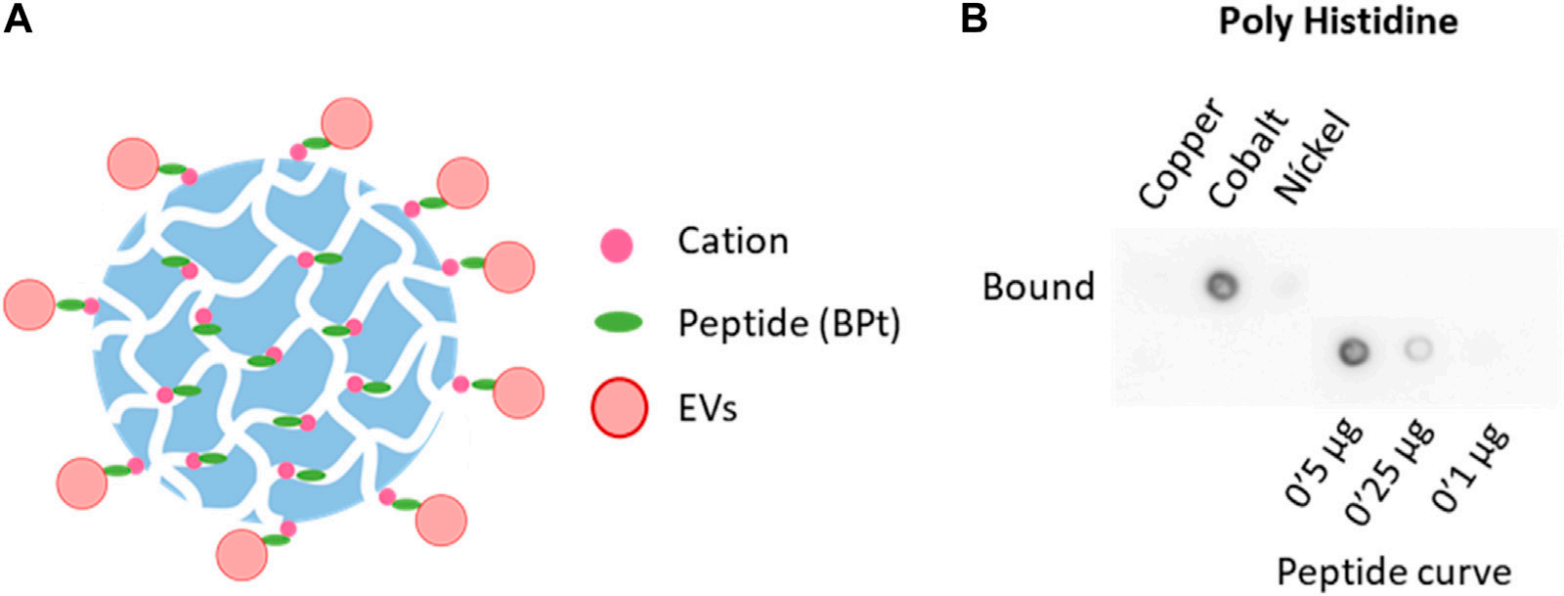
Selection of chelate resin. **(A)** Scheme of peptide (BPt) and EVs binding to cation agaroses. **(B)** Dot blot analysis with anti poly-Histidine antibody to compare BPt binding to copper, cobalt, and nickel agaroses. 0.1 mg of BPt was incubated with 50 µL of copper, cobalt, or nickel agarose for 1 h at RT.

In order to determine if membrane–sensing peptides can be employed for EVs isolation, we designed a strategy for affinity isolation with agarose beads of EVs from concentrated conditioned media. We employed a tandem version of a bradykinin-derived peptide (BPt) that has been previously reported to efficiently capture sEVs on a flat well format (Gori et al., 2020). We modified the peptide to include a 6His tag that could non-covalently bind to divalent cations-carrying agarose resins (Figure 1A).

First, we tested BPt binding to different cation agaroses which differ in the cation employed and cation density (see Table 1 under methods). For these experiments, we incubated 0.1 mg of peptide with 50 μL of the different resins for 1 h at RT, under rotation. Resins were then washed twice with PBS and boiled in a final volume of 150 μL (including resin volume) with Laemmli buffer. Peptide binding was analyzed by dot-blot of 1 μL of lysate sample using an anti-poli-His antibody. As can be clearly seen in Figure 1B, cobalt agarose was the most efficient in BPt binding and chosen for all subsequent experiments.

### Optimization of peptide concentration and incubation times

**FIGURE 2:**
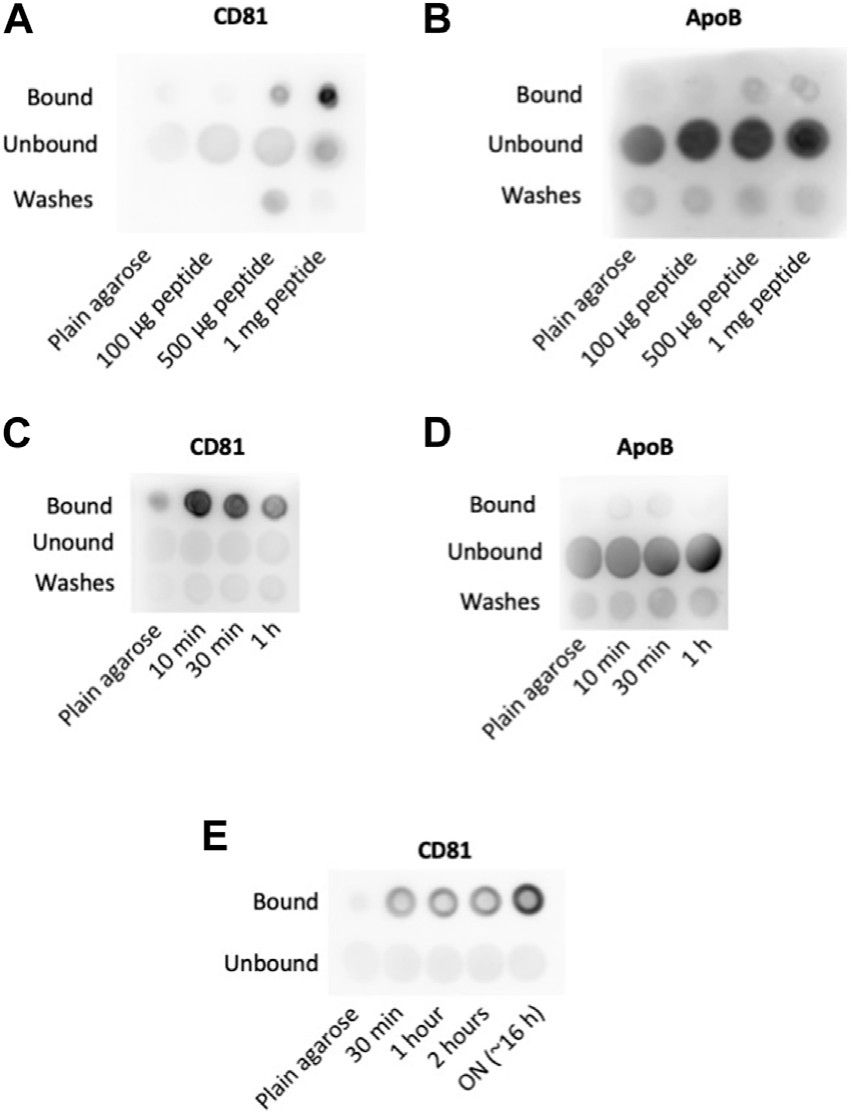
Optimization of concentration and incubation times. Peptide concentration: different amounts of peptide as indicated were incubated with 10 µL of cobalt agarose for 1 h at RT. Then, 100 µL of concentrated conditioned media were added and incubated ON at 4°C. Anti-CD81 (5A6) **(A)** and anti-ApoB **(B)** antibodies were used for EVs and LPPs detection, respectively. Peptide incubation time: dot blot analyses to compare EVs (CD81) **(C)** and LPPs (ApoB) **(D)** binding to agaroses incubated with 0.5 mg of peptide on 10 µL of cobalt agarose for the indicated times before the addition of 100 µL of concentrated conditioned media. **(E)** EV binding time: Dot blot analysis of EV binding changing the times of incubation at 4°C with concentrated conditioned medium. As in previous experiments, 0.5 mg of peptide were incubated with 10 µL of cobalt agarose for 10 min before the addition of concentrated conditioned medium.

To assess whether this strategy was efficient in EV capture and to analyze the amount of peptide required, we incubated for 1 h different amounts of BPt with 10 μL of cobalt agarose. After washing, each sample was incubated ON with 100 μL of concentrated conditioned media from a SKMEL147 culture, washed again with PBS and boiled with Laemmli buffer in a final volume of 30 μL. 1 μL of each sample was analyzed by dot blot using anti-CD81 tetraspanin as EV marker. We observed dose response precipitation of EVs as we increased the amount of BPt, which demonstrated an effective EVs isolation capacity of bradykinin peptide (Figure 2A). As control, we incubated parallel dot blots with anti-ApoB antibodies to assess lipoprotein (LPP) contamination of the EV samples. As shown in Figure 2B, we could not observe binding of lipoproteins to the peptide-agarose, which confirms the specificity of this BPt for lipids on curved membranes as those in EVs, but not on LPPs.

Agarose resins are composed of porous beads of several hundreds of nm in diameter. EVs would not be able to access the pores, but the soluble peptide can well bind to those cations exposed inside the pores. Therefore, EV binding would only be efficient to the molecules of BPt exposed on the agarose resin beads surface but not to those bound to cations inside the pores (Figure 1A). To optimize peptide exposure on the surface of the resins, we reduced peptide-resin incubation times. In these experiments, we incubated 0.5 mg of peptide with 10 μL of agarose resins for different times, in a final volume of 100 μL. After washing the resin, we performed the incubation with 100 µL of the concentrated conditioned media ON at 4°C, in a final volume of 100 μL. As shown in Figure 2C, we could observe an increment in the EV capture when BPt was incubated with agarose only for 10 min. Again, no LPP binding to the resins could be observed (Figure 2D).

Then, we analyzed different incubation times of BPt-carrying resins with concentrated conditioned media for EV binding. We incubated 0.5 mg of peptide with 10 μL of cobalt agarose for 10 min and after washing, with 100 μL of concentrated conditioned media for different times at 4°C. These analyses revealed that overnight incubation was the most optimal timing for this step (Figure 2E).

### Optimization of cobalt resins

**FIGURE 3:**
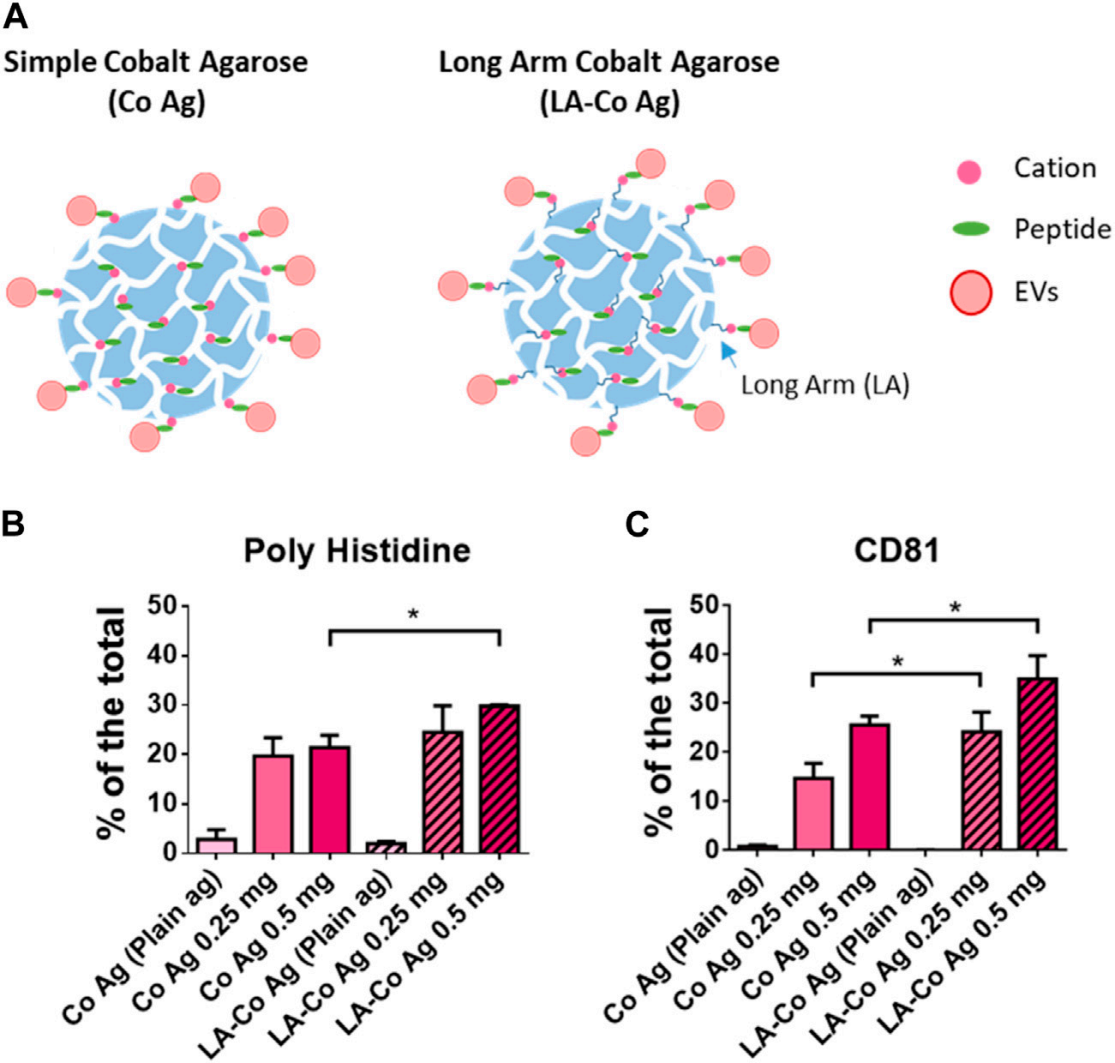
Optimization of chelate exposure. **(A)** Scheme of the two different cobalt agaroses tested which differ in the length of the linker between the agarose surface and the cobalt cation. Dot blot analysis of peptide (poly Histidine) **(B)** and EVs binding (CD81) **(C)** obtained with the two agaroses tested: Simple Cobalt Agaroses (Co Ag) and Long Arm Cobalt Agarose (LA-Co Ag). LA-Co Ag present half of cations than Co Ag. 250 or 500 µg were incubated with 10 µL of both agaroses. 100 μL of concentrated conditioned media was employed for EV binding. Results are represented as the % of a given marker signal in each dot. The means ± SEM of 3 independent experiments are shown. Significant differences in the T-student statistical test are indicated with * (*p* ≤ 0.05).

Since peptide exposure on the surface seemed to be a limiting factor, we decided to compare two different Cobalt-borne resins that differ in the length of the arm that connects the agarose surface to the cobalt chelate, but also in the density of cobalt chelates (Figure 3A), having the resin with the long-arm (12 atoms LA-Co Ag) half of the cobalt chelates (Table 1). Two different amounts of BPt (500 or 250 μg) were incubated for 10 min with 10 μL of both resins (Cobalt agarose and Long Arm Cobalt agarose; Co Ag and LA-Co Ag, respectively). EVs binding was also carried out as in previous experiments. Although the number of binding sites for peptide in LA-Co agarose were lower than in simple cobalt agarose, an increment in the amount of BPt bounded to LA-Co Ag was observed, being even significantly higher using 500 µg of peptide (Figure 3B). When we analyzed and quantitated EV binding, we could also observe a better isolation yield for LA-Co agarose (Figure 3C). These results further confirm that peptide exposure on the agarose beads is a crucial factor for the efficient EV binding. Therefore, LA-Co Ag was employed in subsequent experiments.

### Analysis of different peptide architectures

**FIGURE 4:**
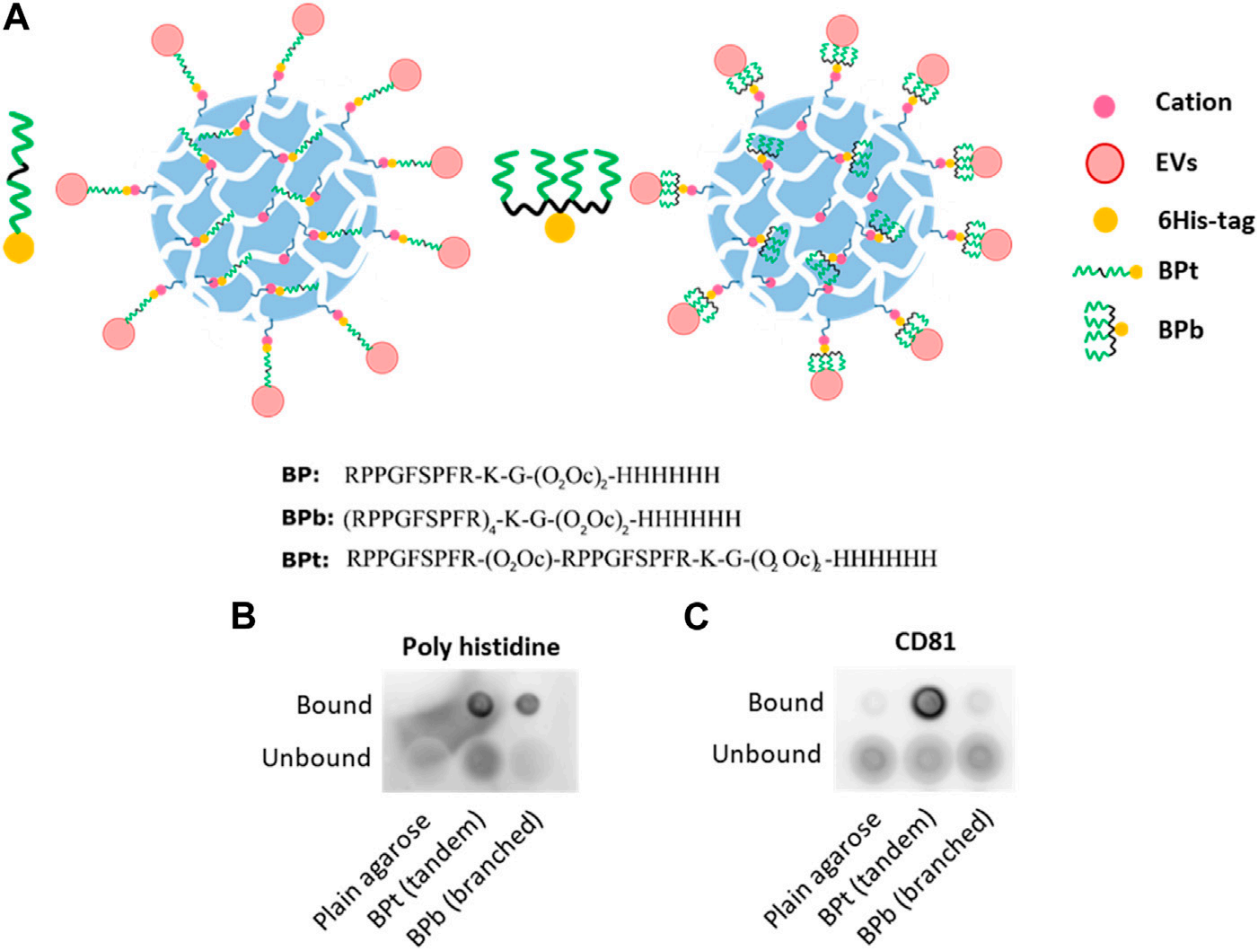
**(A)** Scheme and sequences of the two versions of BP tested and its binding to LA-Co agarose. BPt is a tandem version composed of 2 BP repetitions and BPb is a branched version composed of 4 BP repetitions. Dot blot analysis of peptide (Poly Histidine) **(B)** and EVs (CD81) **(C)** binding when tandem (BPt) or branched (BPb) peptide is employed. Again, 0.5 mg of peptides were incubated with 10 µL of LA-Co agarose before the addition of 100 µL of concentrated conditioned media.

We next decided to compare different architectures of the Bradykinin peptide, including the tandem repeats employed so far (BPt) or a tetra-branched (BPb) (Figure 4A). Multivalent probes were indeed showed to play a favorable role on EV binding efficiency (Saludes et al., 2013; Gori et al., 2020), likely due to cooperative effects that amplify peptide binding affinity (Gori et al., 2017). Using the same amount of peptide (500 μg) for binding to 10 µL of LA-Co agarose (Figure 4B), we observed a clearly higher EVs binding when using BPt in comparison to BPb version (Figure 4C). Thus, we continued using BPt for subsequent experiments.

### Analysis of the impact of pH during isolation procedure

**FIGURE 5:**
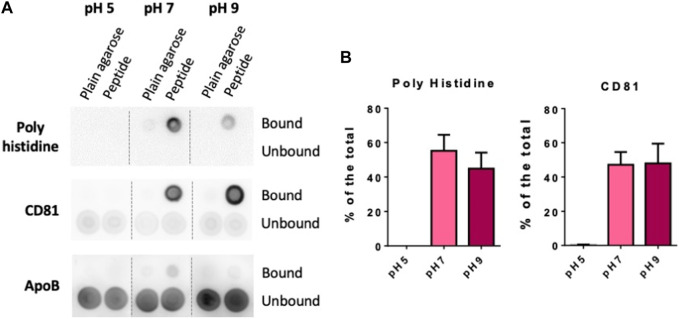
Optimization of pH **(A)** Dot blot analysis of peptide and EV binding at different pH. 0.5 mg of BPt were incubated with 10 µL of LA-Co agarose in a final volume of 100 µL using PBS of different pH. After washing with different pH buffer, 100 µL of concentrated conditioned media diluted by half in PBS at the corresponding pH were added. **(B)** Quantifications of dot blot analysis. Means ± SEM of 3 independent experiments are shown. Significant differences in the T-student statistical test are indicated with * (*p* ≤ 0.05).

Finally, we also tested if changes on buffer pH would affect both the steps of peptide binding and EVs isolation. Again, we employed 10 µL of LA-Co agarose, 0.5 mg of peptide and 100 µL of concentrated conditioned media diluted by half in PBS at the different pH for peptide and EV binding. When experiments were carried out in PBS at pH5, we could neither observe BPt binding to the resin nor EV isolation (Figure 5A). BPt peptide binding to the resins was slightly higher in PBS at pH7 compared to PBS at pH9. In contrast, although the amount of peptide that bound to the resin was lower at pH9, EV yield was in some experiments a bit higher although differences were not significant (Figure 5B), however, the combination of both factors (peptide binding and EV capture) made differences in results between pH7 and pH9 not significant. Subsequent experiments were performed at neutral pH, to facilitate downstream functional analyses.

### EV elution and characterization

Although for many characterization experiments such as proteomics, western blot or RNA analyses, we can directly assess the composition of resin-bound EVs, the His-Tag approach we developed allows for a simple and gentle separation of EVs from the column after purification by simple competition with imidazole or elution with EDTA. Thus, for a more detailed characterization of EVs isolated by BPt affinity method, we eluted EVs from the resins by incubating 40 µL of resin (LA-Co agarose carrying 1 mg of BPt and incubated with 200 µL of CCM) with 100 µL of PBS/0.5 M imidazole. For comparison we isolated EVs from 400 µL of CCM by standard size exclusion chromatography (SEC).

**FIGURE 6:**
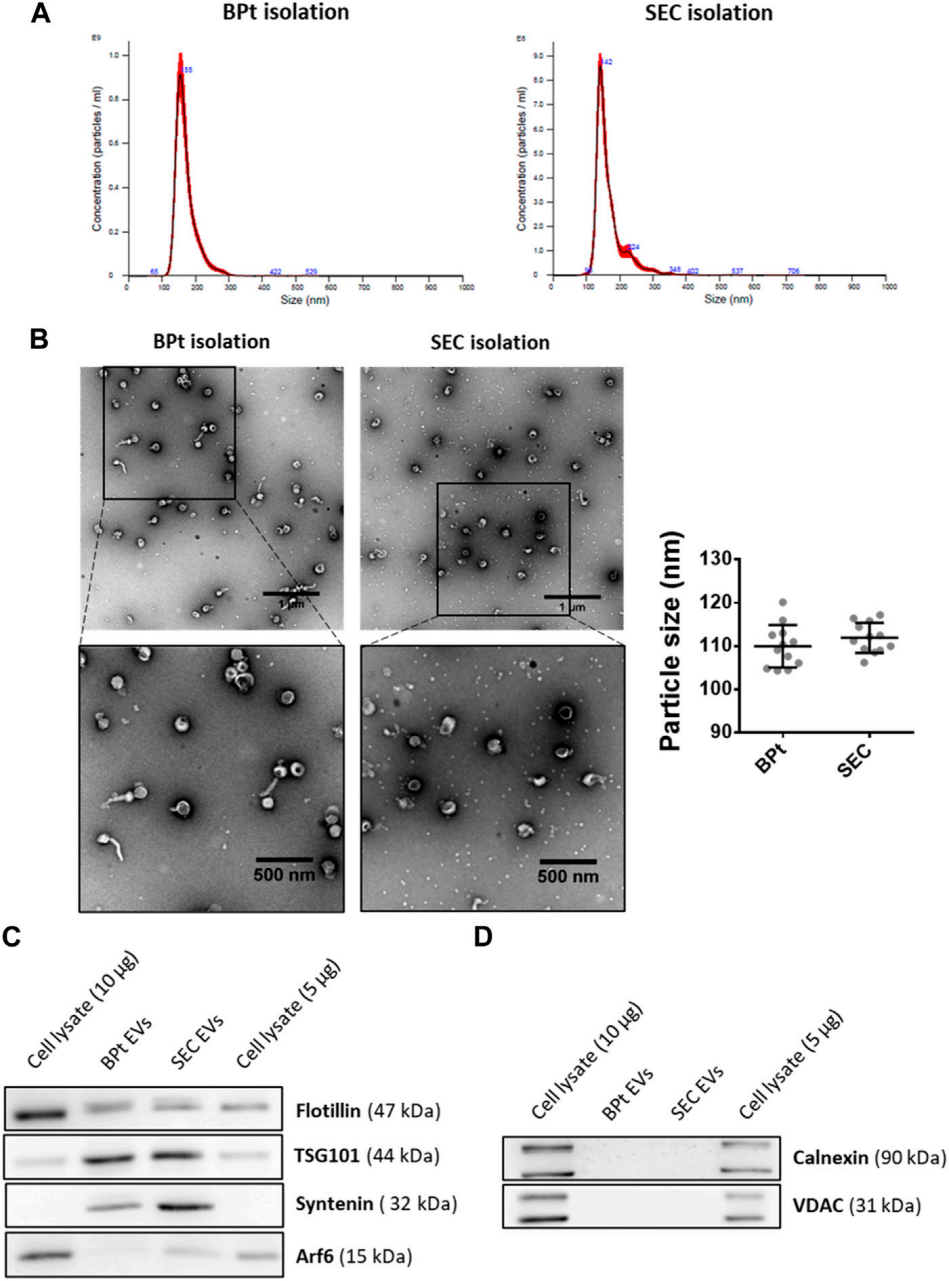
EV characterization. For EV isolation using BPT-based affinity chromatography, 40 µL of LA-Co agarose, 1 mg of BPt and 200 µL of concentrated conditioned media were used. After washing bounded EVs were eluted from agarose by incubation with 100 µL of filtrated PBS containing 0.5 M imidazole. For SEC isolation, 400 µL of concentrated conditioned media were employed for EV isolation. **(A)** Size profile of isolated EVs by BPt affinity or SEC isolation analyzed by NTA. **(B)** Representative TEM images of negatively stained EV samples and analysis of mean EV diameter from TEM Exosome Analyser on TEM images. Scale bars = 1 µm or 500 nm (on close up images). At least 9 images of 3 independent experiments were analyzed for each condition and the data was shown as mean ± SEM. Western blot analysis of **(C)** EV markers (Flotillin, TSG101, Syntenin, and ARF6), **(D)** non-EV markers (Calnexin or VDAC) in EV isolated samples.

EVs size distribution of the isolated EVs was similar to those obtained by SEC from the same CCM, as revealed by both NTA analysis (Figure 6A) and TEM images analysis (Figure 6B). Moreover, we could observe a clear reduction in the amount of LPP particles in TEM images of EV samples obtained by BPt affinity method, in comparison to EV samples obtained by SEC (Figure 6B). We performed western blot analysis normalizing the samples to the number of particles as quantitated by NTA. We could also corroborate the isolation of EVs using BPt, by detection of EV-markers like TSG101, Syntenin-1 and Flotillin (Figure 6C). The absence of non-EV markers like VDAC and Calnexin (Figure 6D) also corroborated proper isolation of EVs.

### Comparison of BPt affinity isolation with commercial tools and kits

**FIGURE 7:**
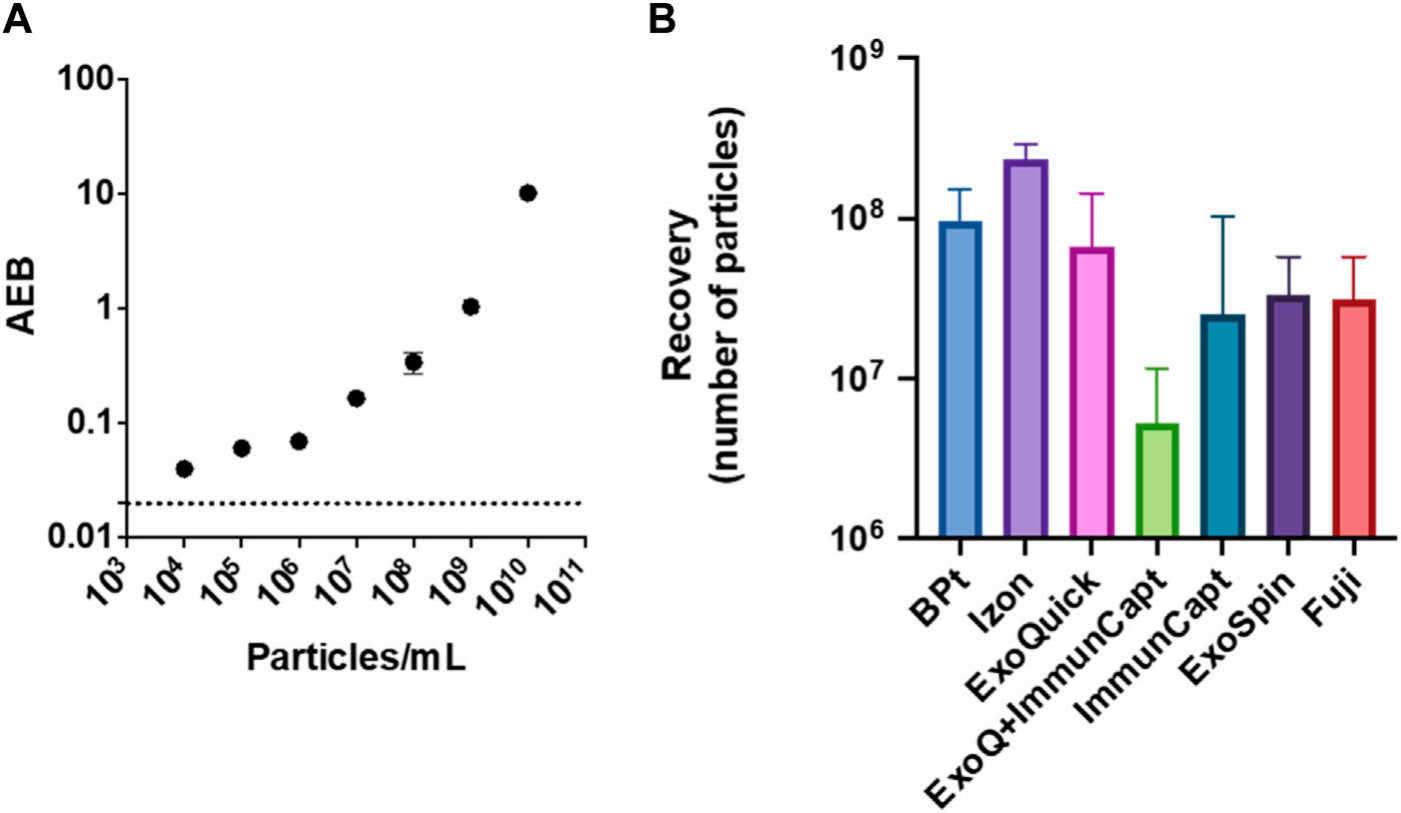
EV yield in comparison to commercial kits. Customized SiMoA (Single Molecule Assay) pan-tetraspanin EV detection was used to estimate EV recovery after BPt protocol and other commercial tools. **(A)** SiMoA results of pan-tetraspanin immune-phenotyping of EVs sample dilutions ranging from 10^4^ to 10^10^ particle/mL, expressed as Average Enzyme per Bead (AEB) **(B)** EV recovery yield after normalization to starting sample volume for BPt protocol, IZON SEC, ExoQuick precipitation, immune-capture on magnetic beads preceded by ExoQuick precipitation, immune-capture on magnetic beads only, ExoSpin kit and phosphatidylserine affinity capture on magnetic beads by FujiFilm. Bar errors indicate SD from 3 technical replicates.

BPt optimized protocol was compared to commercially available tools and kits: size exclusion chromatography by IZON qEV columns, polymer precipitation (Exoquick), bead-based immunocapture by Dynabeads conjugated to anti CD9/CD63/CD81 antibodies (immune-capture was tested alone or after concentration via polymer precipitation), ExoSpin, and bead-based phosphatidylserine affinity capture by MagCapture™ (Fujifilm). Although different starting sample volumes and concentrations were used to comply with manufacturer’s instructions and kit requirements, all different methods allowed to collect particle fractions within 100–200 µL of final volume (see *Materials and Methods*). Customized SiMoA (Single Molecule Assay) pan-tetraspanin EV immune-detection (Frigerio et al., 2022) was selected to provide high sensitivity estimation of EV yield following each isolation method. SiMoA assays were developed according to the Quanterix Homebrew assay instructions as detailed in the Materials and Methods Section. Serial dilutions of the starting EV sample (CM 45x) were analyzed, and a calibration curve establish according to NTA determination of particle concentration in each sample (Figure 7A). Then all EV samples obtained after isolation with BPt and the selected commercial kits were analyzed by SiMoA assay. The raw data obtained were first interpolated in the calibration curve for determination of concentration of pan-tetraspanin positive particles in each analyzed sample, which was then normalized by the volume of the starting sample material to calculate the isolation yield of each method (Figure 7B). EV recovery from IZON columns resulted in the highest yield whereas BPt protocol was superior to all other tested tools and kits.

### Functional analysis of purified EVs

**FIGURE 8:**
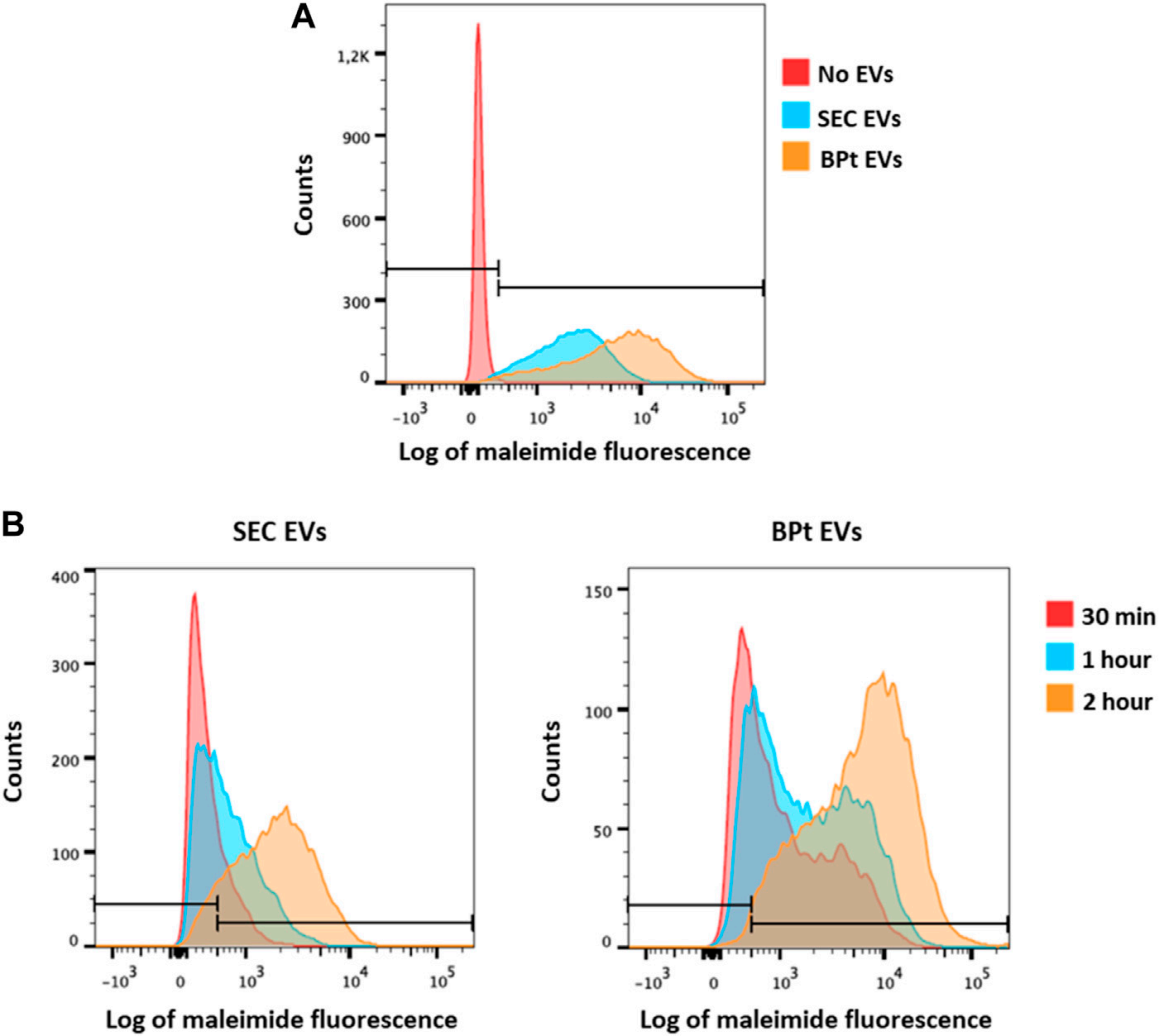
Analysis of EV uptake. SKMEL-147 cells were incubated with 25,000 EVs/cell, previously labelled with maleimide-Alexa633. EV uptake by SKMEL-147 cells was assessed by flow cytometry. **(A)** Uptake analysis of EVs isolated by BPt capture and imidazole elution (orange) or SEC (blue) after 2 h of incubation with SKMEL-147 cells. Fluorescence of the negative control (cells without EVs) is shown in the red histogram. **(B)** Uptake analysis of EVs after incubation with SKMEL-147 cells during 30 min (red), 1 h (blue) or 2 h (orange). EVs were previously isolated by SEC (left plot) or BPt capture and EDTA elution (right plot). Bars indicate negative signal threshold from SKMEL-147 cells without EVs.

As previously mentioned, one of the advantages of the approach here designed is the ability to gently elute EVs from the purification beads and its potential application in subsequent functional experiments. Thus, to assess the functionality of the eluted EVs, we evaluated the capacity of SKMEL-147 cells to uptake EVs isolated using BPt in comparison to EVs isolated by SEC. To this end, EVs were labelled with a fluorescent maleimide compound and then EVs were incubated with SKMEL147 cells before analysis by flow cytometry of EV capture. As observed in Figure 8, EVs isolated by BPt and posterior elution with imidazole (Figure 8A) or EDTA (Figure 8B) were efficiently captured by target cells, which validated their use in functional experiments.

## Discussion

One main limiting factor for translation of EV analysis in the clinical practice for biomarker discovery or validation is the lack of a suitable isolation method that would render high yield and very high purity samples in a quick and easily standardized procedure. These aspects are subjected even to stronger limitations when the starting sample is a complex biofluid in which many different particles that overlap in size, density or other biophysical characteristics coexist. Most affinity-based methods would comply with the high purity requisite but are dependent on the presence on the EV surface of a given target biomarker, thus skewing the isolation yield towards a given subpopulation of vesicles. Here we designed a method based on previous promising results obtained using bradykinin-derived peptide (BP) as membrane-binding peptide for EVs attachment and analysis using a microarray platform (Gori et al., 2020).

For an initial proof of concept to test whether this peptide will be also useful for EVs isolation, we designed a simple modification of the peptide consisting in the addition of a 6His end tag only, with the aim to develop an easy chromatographic method based on agarose beads chemically modified with cation chelates that would specifically bind to the 6His-tagged membrane-sensing peptide. This approach presents several advantages: i) cation-carrying agaroses are widely used and are already standardized for His-tagged protein isolation with good results, ii) the affinity protocol can be performed in small volumes and would be a feasible and manageable method for a clinical routine and iii) elution with imidazole or EDTA allows a gentle and easy elution without EV damage, which should allow subsequent characterization and functional analysis of EVs.

With this strategy in mind, we have optimized all parameters of the protocol starting with the chelate in the resin, the concentrations and incubation times. In these analyses we realized that peptide exposure was a limiting factor. Agarose beads are formed by a network of fibers leading to a symmetric porous structure. The size of these pores is smaller than EVs, which limits EV binding to those BPt molecules present on the bead surface. Results demonstrated a higher binding of EVs when peptide was incubated with the resin for only 10 min. In agreement with this idea using a longer linker to the chelate also improved the EV isolation yield.

Binding site exposure on the peptide was also shown to be better with tandem version of the peptide (BPt), which give better yield when compared to a branched version (BPb), even though the tandem version had two repeats and the branched version 4 repeats of the binding site. All these results suggest a great dependency on the spatial availability of BP for a more efficient EVs binding, which is improved when the distance between BP and agarose surface is increased. These results are in line with previous data on the relevance for peptide probes of strict control of surface exposure to complex biological samples (Gori et al., 2016; Odinolfi et al., 2019). Taking it into account, further improvement of the EVs binding capacity of BP could be obtained using an agarose with an even longer arm between cobalt and the bead surface.

Concerning experiments carried out with buffers at different pH, we could observe two opposite effects. First, we observed a maximum of BPt binding at pH 7, which is in concordance with general cobalt resins recommendations for users (pH 7–8; ABT or GBiosciences). Secondly, results demonstrated an increased EVs binding at pH9, even when peptide is reduced in that condition. As BP recognition and interaction of EVs membrane is firstly based on ionic interactions with phospholipids, it makes sense that increasing pH becomes a higher EV binding, due to an increase in negative charge density of membranes (Stillwell, 2016). However, the weight of these two events (peptide-resin and EV-peptide binding) varied from experiment to experiment so that the final overall yield was similar at both pH.

In summary, our optimized protocol uses 0.5 mg of peptide which is incubated for 10 min with 10 µL of Long-arm Cobalt agarose, and thereafter overnight with 100 µL of concentrated conditioned medium (corresponding to 4·10^5^ initial cells; or ¼ p150 confluent plate after 6 days of EV production) at pH close to neutral. Although this protocol has two independent incubation steps, it can be easily automated and adapted to the clinical practice and requires no more instrumentation than a bench small centrifuge. Modifications of this approach to achieve a covalent binding between peptide and agarose would be possible, further easing the isolation procedure, but would surely imply a harsher elution that may damage EVs.

Many downstream analyses (western blot, proteomics, DNA or RNA extraction, qPCR) can be easily performed adding lysis buffer or nucleic acid extraction buffer to the sample directly bound on the agarose beads. However, EV enumeration and electron microscopy requires EV elution from the resin. Using imidazole or EDTA we could gently elute the sample characterize our affinity isolated EVs and compare them to classical SEC or several commercial isolation methods. We could demonstrate by analysis of size, morphology and protein cargo that using BPt-affinity chromatography we were able to isolate EVs carrying the most common EV markers (detected by both western blot and SiMoA analyses, and devoid of non-EV abundant proteins such as calnexin or VDAC. When analyzing the level of co-isolation of LPPs, we could not observe significant levels of ApoB by dot blot and samples appeared clean from small LPP particles when analyzed by TEM, with higher purity than samples obtained by SEC. This suggest a low presence of contaminants in EVs isolates when using this methodology, which will have to be further corroborated with more complex biological samples (such us serum or plasma), where the levels of LPPs is considerably high clearly outnumbering EVs (Caulfield et al., 2008; Coumans et al., 2017).

Regarding elution, one aspect that should be considered is that imidazole may reduce some disulfide bonds in the sample, which may affect some downstream analyses. In that regard, we realized that recognition of tetraspanin EV markers like CD63, CD9, and CD81 was greatly diminished in imidazole-eluted samples only after denaturation, so that these markers were barely detected by western blot (Supplementary Figure S1A) but properly detected in SiMoA analysis or dot blot without denaturing the sample (Supplementary Figure S1C). This effect was not observed when eluting with EDTA (Supplementary Figures S1B, C).

Regarding vesicle size, comparing BPt-affinity chromatography with SEC, we observed similar size profiles in EV samples by both NTA and TEM analyses. Although we could expected that BPt-affinity would isolate smaller EVs than SEC, as in a previous work this peptide was demonstrated to specifically bind to small EVs (sEVs) (Gori et al., 2020), the cell line SKMEL-147 used in this study secretes rather homogeneous vesicles of a small size. However, when using conditioned media from a lung adenocarcinoma cell line (H3122) which presents a more heterogeneous EVs population in terms of size, we could observe that BPt was able to pull down only the smallest EVs both from the microvesicles subpopulation (enriched by UC at 10,000 g) but had a wide recovery on the 100,000 g pellet enriched the small EVs subpopulation as observed in the NTA profile (Supplementary Figure S2), as expected from the mode of binding of the BPt peptide.

When analyzing EV yield, BPt affinity chromatography also outperformed most other methods and commercial kits, being IZON SEC columns the only method that rendered slightly higher yields than BPt as quantitated on tetraspanin-based detection on EVs. BPt isolated EVs proved to be also suitable for downstream functional analysis, as evidenced by measurements of *in vitro* EV uptake by target cells. In these analyses maleimide labelling of EVs gave better signal on BPt-purified EVs than SEC isolated samples, probably due to the recognition of BPt by maleimide.

In this manuscript we focused on exploiting membrane sensing peptides in EV isolation and in the optimization of the main parameters that could influence this approach. However, the use of membrane sensing peptides in the EV field is not limited to affinity chromatography but may also be combined with other detection techniques such as antibody staining (Gori et al., 2020) or fluorescence polarization technique, as already demonstrated with tetraspanin-targetting peptides (Kalimuthu et al., 2019; Takahashi et al., 2022).

## Conclusion

In summary, this new isolation methodology based on the recognition of general membrane characteristics of EVs can be a good option for a total isolation of EVs without introducing bias based on the surface markers. It can be used in any species EV sample, enabling this approach to samples from animal or plant species against which no suitable antibodies exist. Being an affinity method, the sample handling protocol is very simple, and less time-consuming than traditional methods, does not require specialized equipment and can be easily introduced in a clinical automated routine. This method can also be scaled up or down according to operator needs, with the possibility of analyzing very low amounts of sample for biomarker analyses. Finally, it is compatible with any downstream analyses thanks to the gentle elution procedure.

## Data Availability

The raw data supporting the conclusion of this article will be made available by the authors, without undue reservation.
