# Supplementary material for: Proof of concept of using a membrane-sensing peptide for sEVs affinity-based isolation
Source: Front Bioeng Biotechnol. 2023 Aug 11;11:1238898. doi: 10.3389/fbioe.2023.1238898 (PMC10457001; doi:10.3389/fbioe.2023.1238898)
Supplement: Supplementary file 1 [file Image2.pdf]

Supplementary Figure 2.

sEVs UC

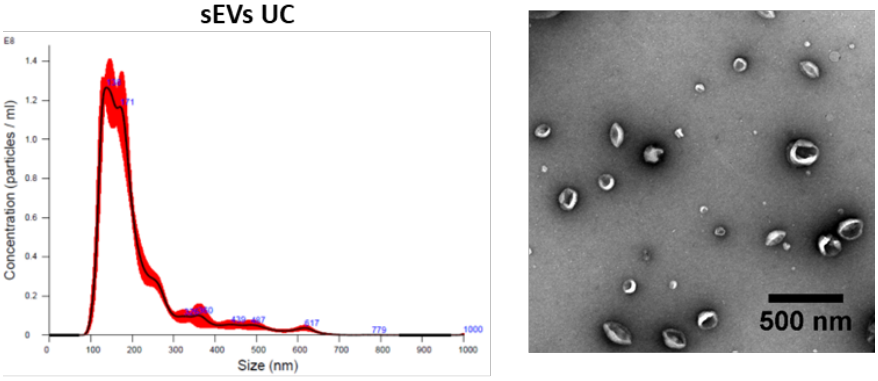

sEVs UC + BPt

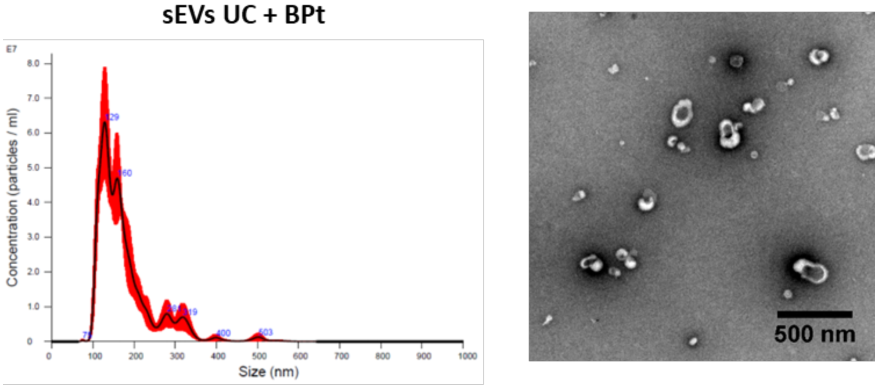

Microvesicles UC

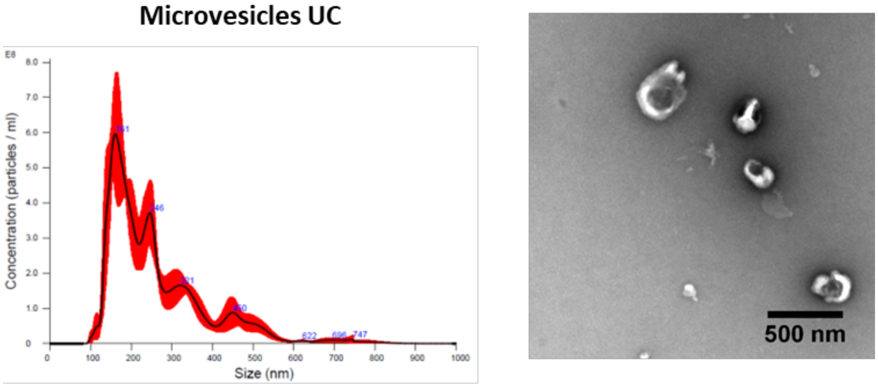

Microvesicles UC + BPt

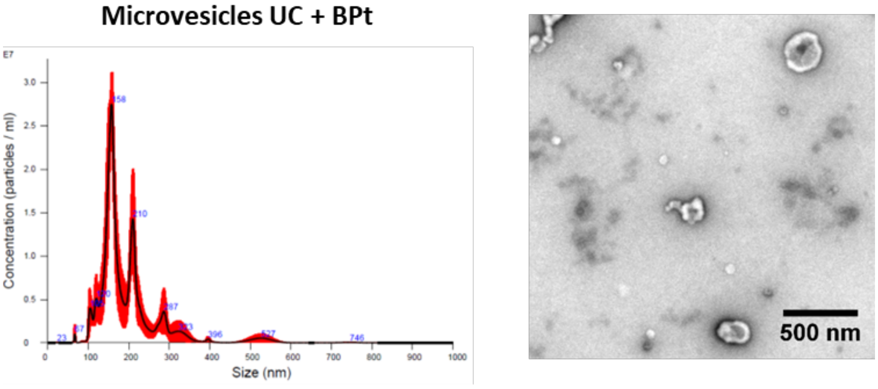

Supplementary Figure 2. Analyses of EVs of different sizes isolated by differential UC followed by BPt.

$2x10^6$ cells from the lung adenocarcinoma cell line H3122 were grown in p150 plates for 5 days for EVs production in RPMI supplemented with 5% EV-depleted FBS, L-Glutamine (1mM), sodium Pyruvate (1mM), non-essential aminoacids (0.1mM), HEPES (10 mM), penicillin (100 U/ml) and streptomycin (100µg/ml), at 37°C in a 5% CO_2 atmosphere. Remaining cells, cellular debris and apoptotic bodies were eliminated from conditioned media similarly to SKMEL-147 conditioned media. Supernatant was ultracentrifuged at 10,000g for 1 h for microvesicles isolation. Supernatant was ultracentrifuged again at 100,000g for 1 h for small EVs (sEVs) isolation. Then, vesicles were isolated by BPt from both subpopulations. Size profile of isolated EVs by UC or UC plus BPt affinity isolation were analyzed by NTA. On the right side of each NTA size profile, representative TEM images of negatively stained samples are shown (Bars=500nm).
