# Supplementary material for: Proof of concept of using a membrane-sensing peptide for sEVs affinity-based isolation
Source: Front Bioeng Biotechnol. 2023 Aug 11;11:1238898. doi: 10.3389/fbioe.2023.1238898 (PMC10457001; doi:10.3389/fbioe.2023.1238898)
Supplement: Supplementary file 2 [file Image1.pdf]

Supplementary Figure 1

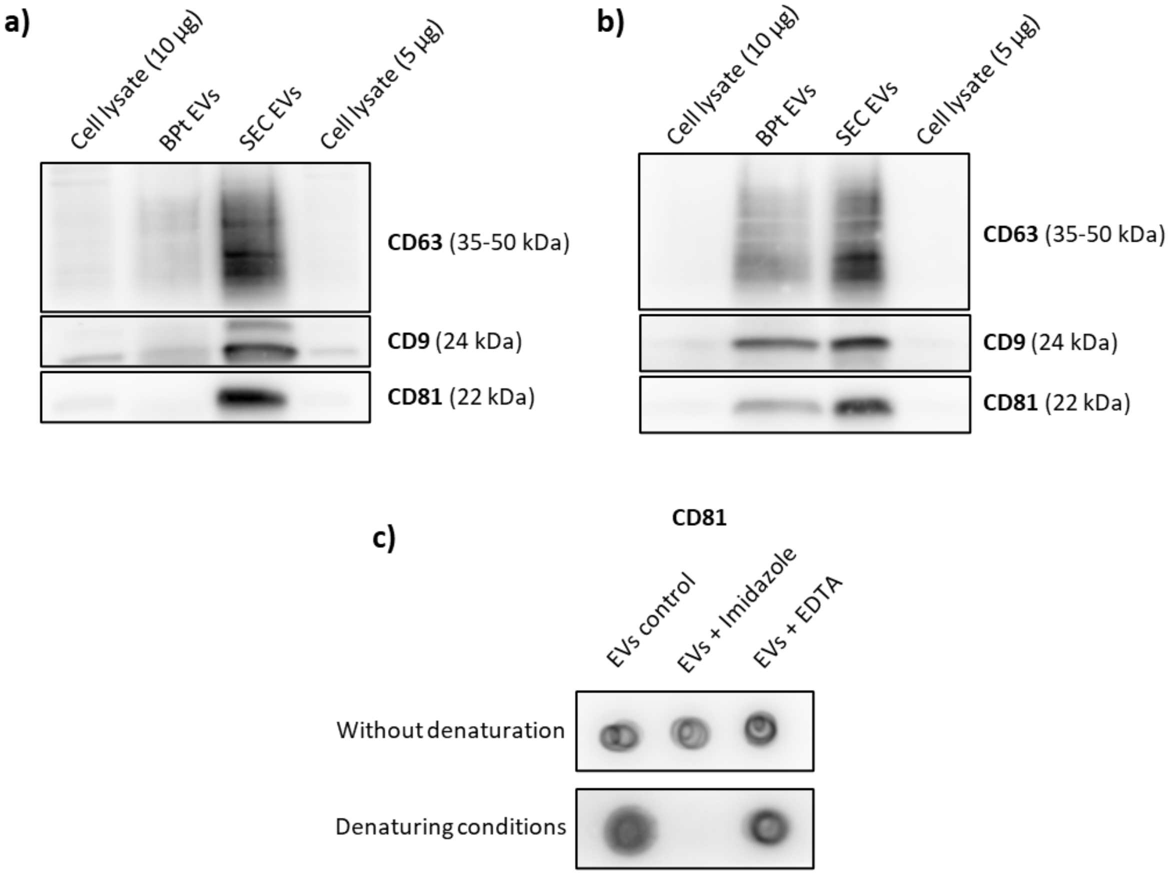

Supplementary Figure 1. Detection of tetraspanin EV markers by western blot and dot blot analyses using different elution reagents. For EV isolation using BPt-based affinity chromatography, 40 µl of LA-Co agarose, 1 mg of BPt and 200 µl of concentrated conditioned media were used. After washing, bounded EVs were eluted from agarose by incubation with 100 µl of filtrated PBS containing **a)** 0.5M imidazole or **b)** 0.1M EDTA. For EVs isolation by SEC, 400µl of concentrated conditioned media were employed. 2 x 10^9 EVs were loaded in each lane after lysis with non-reducing Laemmli buffer. **c)** EVs previously isolated by SEC were diluted 1:1 with PBS (Control), PBS/imidazole 1M or PBS/EDTA 0.2M. 1 µl of each sample was directly (without denaturation) spotted in a nitrocellulose membrane, or after lysis with non-reducing Laemmli buffer and boiling at 96ºC (denaturing conditions), for CD81 recognition by dot blot.
